# A Machine Learning Approach as an Aid for Early COVID-19 Detection

**DOI:** 10.3390/s21124202

**Published:** 2021-06-18

**Authors:** Roberto Martinez-Velazquez, Diana P. Tobón V., Alejandro Sanchez, Abdulmotaleb El Saddik, Emil Petriu

**Affiliations:** 1 School of Electrical Engineering and Computer Science, University of Ottawa, 75 Laurier Ave. E, Ottawa, ON K1N 6N5, Canada; elsaddik@uottawa.ca (A.E.S.); petriu@uottawa.ca (E.P.); 2 Faculty of Engineering, Universidad de Medellín, Carrera 87 No. 30-65, Medellin 050010, Colombia; dtobon@udem.edu.co; 3 Department of Information Technology, University of Colima, Avenida Universidad 333, Las Viboras, 28040 Colima, Col., Mexico; asanchez@ucol.mx

**Keywords:** machine learning, COVID-19 detection, SARS-CoV-2, symptoms-based test

## Abstract

The novel coronavirus SARS-CoV-2 that causes the disease COVID-19 has forced us to go into our homes and limit our physical interactions with others. Economies around the world have come to a halt, with non-essential businesses being forced to close in order to prevent further propagation of the virus. Developing countries are having more difficulties due to their lack of access to diagnostic resources. In this study, we present an approach for detecting COVID-19 infections exclusively on the basis of self-reported symptoms. Such an approach is of great interest because it is relatively inexpensive and easy to deploy at either an individual or population scale. Our best model delivers a sensitivity score of 0.752, a specificity score of 0.609, and an area under the curve for the receiver operating characteristic of 0.728. These are promising results that justify continuing research efforts towards a machine learning test for detecting COVID-19.

## 1. Introduction

The COVID-19 pandemic took the world by surprise. More than 150 million cases have been recorded, while the number of related deaths has surpassed 3 million [1]. As alarming as these numbers are, an argument can be made that the actual number of cases may be higher. To further elaborate on such a claim, we need to understand the concept of “Percent Positive”, also known as the “Positive Rate”, in regard to COVID-19 diagnostic tests. The Percent Positive (PP) is the percentage of positive COVID-19 diagnosis tests from the total tests conducted during a relatively short period of time (e.g., weekly). A high PP can be interpreted in two ways; it could be an indicator that there are high transmission rates among the population, or that the local authorities must conduct more tests. A lower PP is an indicator that there is a lower transmission rate among the population. Assuming that health authorities are implementing effective interventions for reducing spread, transmission rate and PP come hand in hand, because when governments increase their testing capacity in order to lower the PP, more data are available on how the pandemic is spreading, thus providing information necessary for the implementation of such interventions. The World Health Organization has recommended that a PP lower than 5% must be achieved to keep the spread of the pandemic under control. One could argue that widespread testing for COVID-19 in combination with other interventions could lead to lowering the transmission rate of the virus that causes the COVID-19 disease (SARS-CoV-2). In other words, widespread testing is a key element in controlling the pandemic [2,3,4,5,6,7,8,9,10].

Some governments have succeeded at increasing their testing capabilities, while others have struggled at this effort. The understanding of why this happens falls outside of the scope of this work. However, we do know this pandemic took the world by surprise, and healthcare systems and governments were not fully equipped to prevent this disaster, otherwise, we would not be talking about a pandemic. We also know from reported data that some governments conduct COVID-19 diagnosis test on a massive scale, as is the case of the government of United Kingdom, by recording over 2 million tests per million population as of 20 April 2021. Meanwhile, other governments report much lower figures on testing for COVID-19, as is the case of Mexico, with less than 50 thousand tests per million population on the same date [11]. The UK has implemented a strong testing policy, while Mexico has not. On 24 April 2021, the UK reported a PP of 0.2% while Mexico reported a PP of 17.9% as per the live database described in [12] compiling COVID-19 statistics. In this work, we are not going to discuss the reasons behind each country’s testing policy, as it is beyond the scope of this work. However, we observe, from reported data, that Mexico’s scenario is like that of other Latin American countries and some developing countries; even some members of the G20 and G7 have presented a similar scenario at some point or another during the pandemic. One approach that has been adopted to optimize the use of limited testing capabilities is to assess patients to find those who are potentially infected with COVID-19 and to test only those suspected of being infected with COVID-19. The assessment usually requires a health professional to conduct an interview to assess whether a patient may have been infected with the novel SARS-CoV-2 virus. Following the WHO guidelines, countries with such high PP levels and such low COVID-19 testing may need to increase their testing capacity along with a strong diffusion campaign to detect positive cases even in asymptomatic individuals. As counterintuitive as it seems, finding more cases will lead to better understanding of the spread of the virus in local communities. Appropriate interventions implemented by governments will lead to the reduction of local transmission rates and eventually, thus controlling the pandemic [5,13,14,15].

Further elaborating on Mexico’s situation with a low testing rate per million population and high PP, we regard it as unlikely that a country with roughly 130 million people will increase its testing capacity in the near future, especially if health authorities have not done so already. The root of the problem of limited or low testing capacity may find an explanation in the underlying circumstances, which are beyond the scope of this work. However, in such a dire situation, we argue that if COVID-19 testing resources are reserved mainly for suspected positive cases, then a more effective assessment process to decide if a patient is a suspected positive case will help to discover more infections in the community, ideally “asymptomatic” or mildly symptomatic cases. In turn, health authorities will obtain more information on the spread of the pandemic and implement appropriate interventions in the same community.

To decide whether or not a person is suspected of being infected with the virus, an interview with the patient can be conducted by physicians. The compilation of a patient’s reported symptoms and signs during that interview is known as anamnesis. From the data in the anamnesis, the physician can elaborate a hypothesis with a probable diagnosis. This process is known as clinical diagnosis and is heavily dependent on the physician’s skill and experience. During the pandemic, some governments have prioritized access to COVID-19 testing mainly on the basis of signs and symptoms (i.e., clinical diagnosis).

Physicians throughout the Mexican territory have been recording anamnesis data in official databases. We gained access through official means to a subset of the records in the states of Sonora and Tlaxcala.

We analyzed the clinical diagnosis or “probable diagnosis” for each individual in the datasets and compared them to the actual results from diagnostic tests (PCR tests). In a binary classification problem such as predicting whether or not a patient is infected with COVID-19, sensitivity and specificity are useful to better understand the findings of this analysis; these metrics are used to estimate how well a Machine Learning (ML) classifier can generalize, in other words, correctly classify, new data. Sensitivity is the rate of cases correctly classified as positive from the actual number of positive cases in the evaluation dataset. Specificity refers to the rate of instances correctly classified as negative from the total of negative instances in the test dataset. A good classifier will present high sensitivity and specificity, where the model will be capable of correctly classifying new data. We observed in the obtained datasets that physicians in the four states had low sensitivity when identifying COVID-19 positive cases (below 0.4) and specificity below 0.7 from anamnesis, which is only the signs and symptoms reported by the patients. This means that they were better at identifying COVID-19 negative cases than COVID-19 positive cases. Another important metric is precision, which is the rate of predicted positive instances that are correctly classified. The calculated precision for the physicians was less than half. The reason for this is the degree of similarity between the symptoms of a common flu or other diseases caused by different coronaviruses and the symptoms caused by COVID-19. The datasets only had records from people suspected of having an infection, either a flu-like disease or actual COVID-19. They were recommended for diagnostic testing (PCR test). However, if a larger number of people were assessed to find more suspected positive cases (i.e., increasing testing capacity) and a similar sensitivity was present (the same as that present in the dataset for “probable diagnosis”) a high number of individuals infected with SARS-CoV-2 would go undetected in the community (more than 70%), assuming that the limitations in testing capacity persist. Hence, a large portion of the testing capacity would be used to diagnose individuals who are negative for COVID-19, as only half of the suspected positive cases are actual positive cases. Moreover, there are many individuals who avoid being screened for COVID-19 or are simply “asymptomatic” and never realize they are infected, as they do not present the more obvious symptoms [5]. The Mexican government has been widely using a formula for assessment, such as presence of coughing/sneezing, fever or headache, accompanied by any of the following: shortness of breath (more severe cases), sore throat, runny nose, red eyes, muscle or joint pain.

In this work, we train and test several ML models to detect COVID-19based exclusively on symptoms and signs similar to the process of anamnesis assessment. The best classifier among those reported in this study presents a sensitivity and precision higher than health professionals’ “probable diagnosis” or anamnesis; however, the specificity (the rate of negative cases detected from the total negative cases) is lower. The immediate benefit of adopting such an ML-powered assessment approach is to maximize the use of the limited available diagnostic resources, namely PCR tests, to find more COVID-19 positive infections within the community by identifying a higher ratio of actual positive cases (higher sensitivity). A trained classifier may be used implementing an automatic assessment tool, which in turn could be deployed to assess complete populations. This approach would provide more data on the spread of the pandemic, which in turn would enable health authorities to implement interventions to reduce or even suppress infection rates by providing data of potential infections in the community. Another benefit of this approach is that the model uses self-reported symptoms alone to produce a prediction. This means that by leveraging the available mobile devices, it is viable to assess the general population at a very low cost without stretching further diagnostic clinical resources, which may be limited in the community. Therefore, anyone with a smartphone could report signs and symptoms and be automatically screened for COVID-19. This screening approach is cost-efficient and fast, so a larger portion of the general population could be screened multiple times. Additionally, more people infected with SARS-CoV-2 who do not present more obvious symptoms or who present a milder form of COVID-19 could be detected. The affordability of this approach gives developing countries, such as Mexico, a chance to improve their testing capabilities with immediately available tools that are already deployed among the population, such as smartphones.

A Computer-Aided Diagnosis (CADx) system aims to help physicians or health professionals to reach a diagnosis, usually by assisting in the interpretation of medical images, such as X-ray scans or CT scans, among others. More recently, these systems have leveraged Machine Learning (ML) algorithms to segment the images or to detect anomalies related to a health condition. It may be worth clarifying that we are not introducing a CADx system, as the predictions of the classifiers reported in this study are intended to be used for initial assessment and not to support a diagnosis, furthermore, the predictions are based on self-reported symptoms and not images. Nonetheless, a large portion of the reported studies in COVID-19 detection are image-oriented (i.e., chest CT scans or chest X-ray scans), so in the next section, we will address some of these works in order to establish a point of reference for our study.

### 1.1. Previous Work

Since the beginning of the pandemic, researchers around the world have been scrambling to make sense of what is going on. We reviewed some of the most interesting approaches to automatic COVID-19 detection. We identified that the efforts towards automatic COVID-19 detection can be divided into three major groups (i.e., imaging, blood work, and other lab tests, symptoms-based), which are explained as follows.

The imaging group mostly leverages deep learning to detect COVID-19 from chest X-ray scans and/or computerized tomography scans (CT scans). The bulk of the research in automatic detection of COVID-19 fits into this group. In [16], 3D CT scans were used to train a Convolutional Neural Network (CNN) that classifies images to detect COVID-19-related lesion in the lungs with a sensitivity of 0.907 and a specificity of 0.911. In [17], a CNN was trained for COVID-19 lung infection segmentation from CT scans. By segmenting the scans from COVID-19-related lung lesions, the proposed network implicitly predicted COVID-19 infection. The research reported a sensitivity of 0.87 and a specificity of 0.974. Another relevant work is that presented in [18], where lung ultra-sound videos from a total of 35 patients were fed into a CNN to train the network for the automatic detection of COVID-19-related abnormalities in the lungs, reporting an accuracy of 0.96. In [19], the authors adopted a transfer learning approach using a chest X-ray dataset with Xception [20]. Another approach [21] used an unbalanced dataset composed of chest X-ray images and transfer learning with MobileNet [22]. The authors achieved a sensitivity of 0.878 and a specificity of 0.993. Another example of the use of a deep learning approach to detect COVID-19 in chest X-ray images is presented in [23]. The authors presented a slightly different approach, as they used pretrained CNN models to extract features from images (i.e., the output of the final layers in the models). These features were then used to train classifiers with conventional ML models, namely Random Forest, Support Vector Machines and XGBoost. The best ROC reported in this work was 0.99.

In the blood work group, we identified efforts to predict COVID-19 infection based on results from blood, urine, or other laboratory tests. In [24], the authors reported a sensitivity of 0.93 and a specificity of 0.9333, with an SVM classifier trained with a dataset with 18 blood values. Another work [25], reported a sensitivity of 0.75 and a specificity of 0.49 using a dataset with laboratory findings (features) to train an XGBoost classifier. The authors of [26] used the same dataset as that in the previous work; however, the authors used a deep learning approach that yielded a better result on the dataset. In [27], a statistical analysis was conducted to calculate the diagnostic accuracy of different laboratory parameters individually, with an estimated sensitivity >0.9, and specificity <0.4.

Finally, there seems to be limited work on automatic COVID-19 detection based exclusively on symptoms. The authors of [28] used logistic on a dataset with only 64 positive cases of COVID-19 infection. To evaluate the performance of the classifier, the authors used the area under the curve for the receiver operating characteristic (AUC ROC), a metric which is further explained in the next section (Section 2.4. Performance Metrics). In [29], the authors leveraged a hybrid approach by combining wearable sensor data and self-reported symptoms to predict a COVID-19 infection. However, the authors performed a statistical analysis instead of training a classifier. A sensitivity of 0.72 and a specificity of 0.73 were reported in this work. 

Among the most recent efforts, we found one using a combination of voice signals and symptoms, with audio recordings of people coughing (infected and not infected with COVID-19), to train Support Vector Machines (SVM); the authors reported a sensitivity of 0.68 and a specificity of 0.82 [30]. Another interesting example is the work presented in [31], which attracted a lot of attention from the scientific community because the authors discovered that not every patient presents the same symptoms. In fact, they discovered that we could set infected patients into six groups using cauterization on a dataset with 1653 infected participants. However, the authors used the discovered clusters to predict whether or not a patient would require respiratory support, rather than for detecting an infection.

### 1.2. Contribution

Since the pandemic was declared in March 2020, researchers all over the globe have presented several diagnostic tools to help in the efforts to fight the pandemic. It has been established that an effective and fast testing strategy is paramount to the recovery of some form of normalcy, and can help save human lives. A vast majority of this work has been focused on using chest CT scans or chest X-ray scans to train Deep Neural Networks, mainly Convolutional Neural Networks, to assist in the diagnosis of an infection with SARS-CoV-2 (COVID-19). However, due to the constrained availability of diagnostic resources in developing countries, which have been the most affected during this pandemic, an approach requiring CT or X-ray scans would be difficult (at the very least) to scale up and make available for everyone to benefit from the contribution. With the conception of this work, the authors are interested in discovering how well a COVID-19 infection could be detected using self-reported symptoms. In our best results, a voting ensemble of a random forest and a decision tree yielded a mean area under the ROC curve of 0.728, a sensitivity of 0.752, a specificity of 0.609, and a precision of 0.660. We believe that a symptoms-only approach for detecting COVID-19 would be of great interest in terms of practical application. Mexico has over 80 million smartphone users and a symptoms-only assessment technology would enable health authorities to estimate the spread of the pandemic in a large portion of the population, with a higher frequency than is possible with the current testing capacity. As of the submission of this work, there have only been a handful of research examples reported on symptoms-only automatic COVID-19 detection. We attribute this to the lack of datasets reporting symptoms in patients infected with SARS-CoV-2 (COVID-19). The work reported in [28] used a linear regression algorithm, and their dataset contained a relatively small number of positive COVID-19 instances. In the present work, we report results of a wide range of experiments with different classifiers. Our intention is to advance the state of the art in symptoms-only COVID-19 detection so that future experiments can use our results to advance the state of the art in this topic.

It is difficult to find benchmark datasets recording COVID-19 patients and symptoms because of the nature of the data itself and the relatively short period of time since the first case was detected. In that sense, we also contribute by providing a live version of the dataset, which will be updated as soon as health authorities make more data available to us. This is the first dataset of its nature, and includes more than 886 recorded cases of positive SARS-CoV-2 (COVID-19) infection in the Mexican population. Each one of the cases was recorded from in-person interviews at points of care in two different states of the Mexican territory. It is also noted that the dataset is updated with every new dataset that is made available to us by health authorities in Mexico.

### 1.3. Organization of the Paper

The remainder of this paper is organized as follows. Section 2 introduces the reader to a set of basic concepts necessary for the discussion of the experiments as a sort of “common ground”, so that almost any reader can follow the discussion. Next, we continue to explain the methodology we adopted to conduct the study and the criteria we used to evaluate the performance of each one of the classifiers we present. Then, in the same chapter, we proceed to explain the dataset and its features, as well as the variables used for automatic detection of COVID-19. As with any other machine learning-oriented study, we determine and explain beforehand the metric used to evaluate the overall performance of the different classifiers presented in this study. In a dataset with a relatively high dimensionality with respect to the independent variables, some variables “contribute” more than others to the value of the dependent variable. In the same chapter, we present a feature importance analysis to better understand the role of each symptom in detecting COVID-19 in any given case. The document then proceeds to set baseline scores to compare the results of the presented classifiers before and after hyperparameter tuning; the search strategy and the search space for the optimal hyperparameters in this study are also explained in Section 2. Section 3 is dedicated to presenting the results after tuning the classifiers and showcasing the best results with the defined settings. A discussion of the results is presented in Section 4. Section 5 presents the final conclusions for this study and outlines future work in this area.

## 2. Materials and Methods

In this study, we train and evaluate 15 different classifiers with learning algorithms already implemented in scikit-learn [32], namely decision tree (DT), neural network (NN), and support vector machine (SVM). The algorithm used to train the SVM classifier is the C-Support Vector Classifier, an implementation of libsvm for scikit-learn. In addition, we also use two types of ensembles, namely random forest (RF) and a voting classifier (VOTING). An ensemble aggregates the predictions of two or more trained ML models in order to create a prediction on its own, this is why a trained ensemble is considered a model too; while a random forest aggregates the predictions of several decision trees (randomly grown), a voting classifier aggregates the predictions of any type of ML model. In this study, we explore the use of a voting classifier that aggregates the predictions of the decision tree, the neural network, the support vector machine, and the random forest in all possible variations with 2, 3, and 4 votes. The experiments were run on a computer with an Intel core-i7 processor with 2.6 GHz, 16 GB of RAM, and a GPU with 640 CUDA cores. As for the datasets, the public health authorities from the states of Sonora and Tlaxcala in Mexico compiled two different datasets from medical records collected from patients suspected of being infected with SARS-CoV-2. Each state compiled their datasets from their patients separately, but using the same variables. The two datasets were merged and then processed to obtain a single dataset with 1772 complete samples of data collected from 886 COVID-19-positive patients and 886 COVID-19-negative patients. We explain the details of the data collection, preprocessing, feature selection, training, and validation approach in the following subsections.

### 2.1. Machine Learning

#### 2.1.1. Classification Task

Machine learning problems can be classified into two main types of problem: classification and regression. In the following, we explain what classification is in the context of machine learning. For the purposes of this study, we limit our understanding of the concept of classification. In machine learning, a group of similar objects is known as a class, and it is designated with a label. There may be more than one class in a dataset. The problem of classification essentially consists of assigning a label to new objects based on previous observations. Take, for example, a child learning to identify animals. When the child sees a dog for the first time ever, the child learns the name of the animal after the parent explains it; after a few more encounters, and under the supervision of the parent, the child learns that most dogs bark, have four legs, and possess other characteristics or features common to all dogs. The next time the child sees a dog, the child will be able to assign the label “dog” to the animal on its own. The role of the child in this example is the same as the role of a classifier learning to assign labels to new objects from previous observations that are fed into the learning algorithm in the form of datasets [33]. A formal and detailed definition for the classification problem is not necessary to follow the discussion in this study, and is beyond its scope, so we restrict ourselves to offering a simpler explanation of it; the same applies for the rest of the concepts introduced in this section.

#### 2.1.2. Decision Tree

Breiman et al. [34] introduced the decision tree learning algorithm in a study titled Classification and Regression Trees (CART); the learning algorithm has the same name. The CART algorithm follows the divide-and-conquer strategy, growing a tree by splitting the training dataset into as many branches as there are classes found in the target variable. This process is repeated recursively with each branch until a maximum depth has been reached or a split generates pure leaves. At each split, the CART algorithm decides what the best feature is to make the split on, and this decision is based on the purity that a possible split would produce for each feature in the dataset. The purity of a split is a measurement of the homogeneity of each branch (produced from the split). For a binary classification problem, CART grows a binary tree. In this study, we introduce the problem of SARS-CoV-2 detection from self-reported symptoms, which is a binary class classification problem. If applied to this problem, CART would find the symptom that produces the most homogeneous split in the dataset to produce two branches. Let us imagine that the symptom that provides the best split is fever; in this case, those examples who have a fever will be assumed to be infected while those who do not have a fever will be considered infection-free. The CART algorithm recursively grows more branches from that point, and for the next level, it will take other symptoms to do more splits. The key is to find the feature that produces the most homogeneous separation; this is assessed by measuring the impurity of the division. For this purpose, the Gini index or Gini impurity function is used.

The Gini index is a measurement that indicates the probability of a particular sample being misclassified with a given split. The CART algorithm uses the Gini index to decide the best split for growing the branches of the decision tree. For example, if there is a dataset with three independent variables or features and two possible classes to be assigned to a sample, CART will measure the impurity of a split made with each of the three features and select the one that produces the least impure division. The following formula corresponds to the Gini index,

(1) $$Gini\;Index=1-\overset{n}{\underset{i=1}{\sum }}{\left(p_{i}\right)}^{2}$$

where *p* is the probability of a sample being assigned to a particular class i. The learning algorithm measures the Gini index and selects the feature that creates the split with the lowest impurity or misclassification probability, as calculated on the basis of the Gini index, for each branch and each level of the tree.

#### 2.1.3. Artificial Neural Network

Neurons are the building blocks of neural networks; a neuron has input values that are added up and fed to an activation function to generate an output. Each input has a weight that amplifies or reduces an input value. Depending on the application, a neuron may adopt different activation functions, namely threshold function, rectifier, rectifier linear unit, hyperbolic tangent, and sigmoid function. The mathematical definition for a neuron is

(2) $$y=\varphi \left(\overset{m}{\underset{i=1}{\sum }}w_{i}x_{i}\right)$$

where y is the output value of a given neuron and is calculated by evaluating the activation function *φ* on the sum of all weighted inputs w·x. The neural network is an array of neurons organized in layers, which are composed of any number of networks. In some cases, when a neural network has a large number of layers, it then becomes a deep learning problem. The outputs of a layer are inputs for the next layer. It is usually preferable to use the sigmoid function as an activation function, as it has a codomain of real numbers 0 < y, making it possible to adjust the classification threshold and obtain optimal performance. The sigmoid function in the scikit-learn library is implemented as follows:

(3) $$\frac{1}{1+e^{-x}}$$

The weights in a neural network are adjusted to amplify or reduce the effect of a neuron in the output of the final layer, which is accomplished by using a solver or weight optimization algorithm, with the most popular being “adam” [35].

#### 2.1.4. Support Vector Machines

Support vector machines (SVM) are a simple yet powerful machine learning approach. The intuition of SVM is to map each sample in the dataset to a multidimensional space by assigning each feature to a dimension. Thus, the independent variables are also known as dimensions in the SVM. The next step is to define a boundary delimiting the region in the space that corresponds to each class in the dataset. This boundary has the form of a hyperplane in the multidimensional space. Mathematically speaking, a hyperplane is a line, a polynomial function or a radial function, and is used to decide if a new sample belongs to a class or not depending on where in the space it is mapped.

#### 2.1.5. Voting Ensembles

A voting classifier is an ensemble of two or more estimators (classifiers). Each estimator issues a prediction for all classes. In a binary problem such as the one described in this study, each estimator predicts the probability of a positive as well as a negative class. In cases where a hard vote is configured for the voting classifier, the final prediction will be based on a majority vote. However, each estimator is assigned a weight, so it may be the case that each estimator contributes differently to the final prediction. When the voting classifier is set to a soft vote, the final prediction is the result of applying an arg max function to the probabilities of each estimator.

### 2.2. Methodology

This study was conducted in seven major phases: data acquisition, data curation or preprocessing, algorithm selection, determination of performance criteria, feature importance analysis, determination of baseline scores, and hyperparameter optimization.

The beginning of the pandemic was too recent to consider the use of what is known as “benchmark” datasets. However, we did consider finding publicly available datasets from official trusted sources with recordings of symptoms from patients infected with COVID-19. To the best of our knowledge, there are none from a Mexican population. The first phase (data acquisition) consisted of obtaining datasets with recordings from assessments happening at point-of-care centers between December 20, 2019 and June 29, 2020. The datasets were obtained separately from two different government health jurisdictions. The next phase consisted of data curation and preprocessing to join the two separate datasets into a single dataset. After phase two, the resulting dataset had roughly 886 positive records without missing data. In phase three, we selected the learning algorithms to train our classifiers. 

Deep Learning (DL) is the current trend in machine learning, especially for image classification, where Convolutional Neural Networks (CNN) are the de facto standard among the academic community because of the notoriously high performance of CNNs in all their variants in combination with the approach of transfer learning. This could explain why most of the efforts in this problem have been focused on images. However, in our specific case, due to the nature of the features (symptoms) in our dataset, which are dichotomic, we considered that our study would not benefit from the DL approach for the moment, as we would need to find the best way to map each of the records in the dataset onto an image in order to study the problem from an image perspective. Another deterrent to the use of DL was the fact that we had only so many records of positive COVID-19 infections. In this situation, the use of DL is risky, as the network may not be able to correctly generalize from there, and instead it could overfit, thus yielding performance metrics that would drop when tested against new data. However, to cover all bases, we did consider using a fully connected Neural Network (NN). 

We also decided to explore Decision Trees (DT) because of previous success in CADx and eHealth. Again, the binary nature of the features in our dataset played an important role in including DTs in our study, as the metric used to calculate the purity of a branch would be easier to calculate along with the split values (for obvious reasons), which may facilitate training and improve performance. We also chose to include the Support Vector Machines (SVM) learning algorithm in the study due to its known versatility and high classification power in other studies. However, we were aware from the beginning that the class clusters (if present) would be concentrated at the extremes of the multidimensional space (because of the binary nature of the features). 

In this study, we decided to evaluate the classifiers on the basis of how well they could correctly detect infected people (sensitivity), how well the classifiers detected people who were not infected with SARS-CoV-2 (specificity), and finally on what percentage of the predicted positive cases were correctly classified. The logic behind this approach (instead of the accuracy of printing a confusion matrix) is that in this particular problem, it is better to find as many infected people as possible, rather than finding otherwise healthy people. In the case of false positives (if sensitivity is found to be low) one can always ask them to follow official guidelines without risking augmenting the infection rate. In addition, just to have an idea of how many false alarms this approach could be yielding, we also determined the precision of the classifiers. Although we calculated accuracy for all the classifiers, we come close to disregarding this as a way of measuring classifier performance, mainly because we noted from different works that imbalanced datasets were used, meaning that while the reported accuracy may be high, the sensitivity may be low, which in this particular case could be dangerous. The Area Under the ROC Curve is in standard use in these types of studies, as it gives an idea of how capable the classifier is of generalization when moving the classification threshold; meanwhile, the F1 score is a metric that describes how well a classifier can predict positive values without raising false alarms. 

In phase four, we performed a feature importance analysis to determine how much each feature contributed to the classification; some features may introduce what is known as “noise” into the dataset; in other words, we were interested in defining whether some symptoms or sets of symptoms were more important than others in detecting a positive infection. This is of particular interest, as it would help to improve the performance of the classifier if it were to be trained with the more “important” features. In phase five, the baseline scores for the classifiers were determined, and the results from the three learning algorithms and their ensembles were contrasted in preparation for phase six. An important remark is that Section 5 presents the results from the classifiers trained using the same learning algorithms, with all of the hyperparameters set to default values in scikit-learn, in other words, not optimized. Phase six implemented a random search strategy to find the best hyperparameter configuration for the learning algorithms within their respective search spaces. 

### 2.3. Datasets

A successful classifier is one that can generalize the results so that new samples can be correctly classified. The quality of the dataset plays a significant role in the model’s capacity to generalize. In this study, the data came directly from clinics and hospitals in the public health system in the states of Sonora and Tlaxcala in Mexico. As the data were collected by health professionals, there is an assurance that the collected data are accurate, and that the collection process followed the medical guidelines in place for health practitioners in clinics and hospitals in Mexico. Since both datasets were collected following medical guidelines standardized throughout the country (Mexico), we were able to merge them into one single dataset. The public health authorities in both states collected several variables for each sample. These variables included 20 symptoms, comorbidities, signs like sudden onset of symptoms, and laboratory test results for RT-PCR for SARS-CoV-2. Being able to predict a positive infection based on self-reported symptoms is paramount to removing the need for all suspected patients to visit a laboratory to provide samples just to get screened, as the collection of self-reported symptoms can be performed remotely, thus preserving the limited testing capacity for highly suspected cases. We selected the variables corresponding to symptoms in the merged dataset as features for training and validating the model. Additionally, we also selected the variables reporting a sudden onset of symptoms as well as known contact with confirmed cases as features for training and validating the models.

The combined dataset had 10,722 different medical records, accounting for the same number of patients (i.e., one per patient). The 10,722 samples also included patients confirmed to be infected with influenza or coronavirus (i.e., other than SARS-CoV-2). We only kept the samples with a negative result (to any infection) and those affected with SARS-CoV-2. As mentioned, the focus of this study was to explore the use of machine learning techniques to detect COVID-19 based only on self-reported symptoms and signs. We identified 22 features in the dataset that fit this criterion; 20 were symptoms, while the other two features were (1) any known contacts with confirmed COVID-19-positive cases and (2) the sudden onset of the symptoms. The full list of features is presented in Table 1. Features corresponding to symptoms were part of the initial assessment interview conducted at points of care regardless of how advanced the infection may be, and were not proposed by the authors of this study. It was health authorities that deemed there to be value in collecting these symptoms. After identifying these features as independent variables, we removed all incomplete records, i.e., those with empty or unknown values in any field of the 22 variables. The target variable was the result of the RT-PCR test. After undersampling (randomly selecting a subset of noise-free negative records from the dataset to match the number of positive cases) the majority class (negative cases), the resulting dataset had 1772 samples of data from 886 COVID-19-positive patients and 886 COVID-19-negative patients. The result of this process is a noise-free dataset that has many high-quality records for training and validation. Due to the limited number of complete records, we decided to use a 10-fold cross-validation approach in all experiments. 

### 2.4. Performance Metrics

In a binary classification problem such as the one addressed in this study, true-positive (TP) is the number of true instances that are correctly classified by the model. False-negative (FN) is the number of true samples that are misclassified by the model. True-negative (TN) is the number of negative samples that are correctly classified by the model. Finally, false-positive (FP) is the number of negative instances that are misclassified by the model.

Sensitivity is the rate of true-positive instances that are correctly classified in comparison with the total number of true instances. The formula for calculating sensitivity is as follows:

(4) $$Sensitivity=\frac{tp}{tp+fn}$$

Specificity is the rate of true-negative instances that are correctly classified by the model when compared with the total number of negative samples, as follows:

(5) $$Specificity=\frac{tn}{tn+fp}$$

Precision is the rate of true-positive samples when compared with the number of instances classified as positive. The formula is as follows:

(6) $$Precision=\frac{tp}{tp+fp}$$

In many classification problems, it is important to have an idea about how the sensitivity of the model compares to precision. We can do that using the F1 score. The F1 score can have a value between 0 and 1. A perfect F1 score means that all samples classified as positive are, in fact, positive (i.e., no false-positives) and that all positive samples in the dataset are correctly classified by the model (i.e., no false-negatives). Ideally, this is achieved when the model is properly trained. The formula is as follows:

(7) $$F1=\frac{2\times \left(sensitivity\times precision\right)}{\left(sensitivity+precision\right)}$$

Accuracy is the rate of correctly classified samples compared to the complete dataset. Gauging the performance of a model using accuracy by itself can be misleading, as the model could have a high specificity and a low sensitivity, if it is poorly trained. The formula is as follows:

(8) $$Accuracy=\frac{tp+tn}{tp+tn+fp+fn}$$

An ML classifier functions by predicting the probability that a data point has of belonging to a specific class. If a classifier predicts a probability equal to or higher than a threshold value for a data point being in a specific class, then the prediction is settled as positive for that class. If the predicted probability for a data point belonging to a certain class is lower than the threshold, then the prediction is settled as being negative for that same class. By default, the threshold is set to 0.5. In some problems, it is more important to achieve a higher true-positive rate (sensitivity), while having a lower true-negative rate (specificity) is acceptable. In these cases, the threshold for classifying certain data points that otherwise would be classified as negative as positive can be lowered. On the other hand, the threshold can be increased to increase the true-negative rate while lowering the true-positive rate. This is precisely how the receiver operating characteristic (ROC) curve is plotted. The true-positive rate and false-positive rate (1-specificity) are calculated for the classifier at different thresholds. By calculating the area under the ROC curve, the quality of a model can be assessed, and the manner in which the classification thresholds can be played with so that the model renders the best scores for a particular classification problem and circumstance can be understood. The area under the ROC curve (ROC_AUC) was used to assess the performance of all the models reported in this study. Ideally, a properly trained model would get close to 1. The classifier that has the highest sensitivity with the lowest false-positive rate is the best.

### 2.5. Feature Importance Analysis

Feature importance is an impurity-based measurement that represents how important a feature is for predicting the target variable. The Gini index is used to calculate the importance of all the features in the dataset, which is why this technique is also known as Gini importance. Gini importance is calculated by fitting the dataset to a decision tree classifier. Ensembles based on decision trees can calculate feature importance as well. In this study, we calculated the feature importance for the 22 features. The results are shown in Table 1, where each feature from the dataset is ordered by importance in descending order (column ranking).

The Centers for Disease Control and Prevention (CDC) report the symptoms of COVID-19 to be fever, chills, cough, shortness of breath, headache, fatigue, muscle or body aches, loss of smell or taste, sore throat, congestion, diarrhea, and vomiting, although these are in no particular order of importance for diagnosis [36]. The same symptoms are presented in Table 1. One approach to feature selection is to select the features with the highest Gini importance scores and leave out the rest. However, if we adopt that approach, we will leave out some of the symptoms associated with COVID-19 by the CDC. Thus, we propose a different approach for finding the best features to train our machine learning models. We have mentioned that, ideally, a model would predict 100% of true-positive cases with 0% false-positive cases. However, that is not realistic, and we have to aim for the highest possible true-positive rate and the lowest possible false-positive rate; in this case, detecting as many real cases as possible of infected patients with the lowest possible rate of false alarms. The area under the curve for the receiver operating characteristic (ROC_AUC) is the ideal metric to use in these types of cases. We fitted the dataset to a baseline random forest classifier by gradually including the features according to their importance ranking. We selected fever alone to train and validate a classifier, and the ROC_AUC for that model was 0.6328; then we used the top two ranked features to train a new model, which had an ROC_AUC value of 0.6510. We repeated this process until all 22 features were included, and the 22 models were validated. For each iteration, we used 10-fold cross-validation. We can observe from Table 1 (column behavior) that some features cause the ROC_AUC value to drop (even if only a small fraction) when included. We propose removing the variables that reduce the ROC_AUC from the dataset, and only keeping the 14 features that increase the ROC_AUC, namely 1, 2, 4, 5, 6, 8, 11, 12, 13, 14, 17, 18, 20, and 22 from Table 1.

### 2.6. Baseline Scores

Both versions of the dataset have the same 1772 samples, each with 886 SARS-CoV-2-positive patients and 886-negative patients. We selected four learning algorithms to train against the datasets, namely decision tree, neural networks, support vector machine, and random forest. A voting classifier can outperform the classifiers that are part of it, so we explored all possible combinations of two, three and four votes. Table 2 shows the baseline scores for the 15 different resulting classifiers. The baseline hyperparameter configuration for the classifiers is the default configuration, as defined in scikit-learn [32]. The voting classifier built with the baseline NN and SVM report a ROC_AUC score of 0.716.

### 2.7. Hyperparameter Tuning

In this section, we explain our approach to hyperparameter tuning for the 15 classifiers. After tuning each model from the baseline configuration, we evaluated each one with 10-fold cross-validation against the full dataset (22 features) and the reduced dataset (14 features). The results of the tunning process are shown in Table 3.

There are different approaches to searching for the optimal hyperparameter configuration for training a machine learning model, namely particle swarm optimization (PSO), Bayesian optimization (BO), genetic algorithms (GA), grid search (GS) and random search (RS). The grid search method consists of defining a range of all possible values for a set of hyperparameters and testing all possible permutations to find the best configuration to train a machine learning model. This approach guarantees finding the optimal configuration of hyperparameters for a particular learning problem. All of the possible permutations of different values for a defined set of hyperparameters are known as the search space. The downside of grid search is that training a model with the entire search space of hyperparameters is computationally expensive and, in most cases, impractical; the simplicity of its implementation is its primary advantage. An alternative to this is random search. This method selects a random distribution of the entire search space of hyperparameters to test as candidates. This approach does not guarantee finding the optimal solution for a particular problem, but a close-to-optimal solution [37]. We adopted a random search to find a close-to-optimal hyperparameter configuration for the decision tree, neural network, support vector machine, and random forest classifiers. We opted to use a grid search for the voting classifiers.

After optimizing the first four classifiers, we implemented a voting strategy using combinations of the four tuned classifiers (i.e., random forest, neural network, support vector machine, and decision tree). We adopted a grid search strategy to optimize voting classifiers. Table 4 defines the search spaces for all of the classifiers we explored in this study within a delimited set of hyperparameters.

## 3. Results

As shown in Table 2, the baseline ROC_AUC score for the decision tree classifier was 0.622 when trained with the 14-feature dataset, and 0.606 when trained with the dataset with 22 features. After hyperparameter optimization using random search, the ROC_AUC score increased to 0.686 for the 14-feature dataset and 0.693 for the 22-feature dataset, as shown in Table 3. The random search was performed on 0.3% of the search space, as shown in Table 4, which means that we tested 5346 different configurations of hyperparameters. The baseline ROC_AUC for the random forest classifier was 0.68 when trained with the 14-feature dataset and 0.703 with the 22-feature dataset, as shown in Table 2. Table 4 shows the search space for hyperparameter tuning, the ROC_AUC for the 14-feature dataset was 0.723 and 0.730 for the 22-feature dataset, as can be observed in Table 3. The neural network classifier had a baseline ROC_AUC of 0.712 and 0.690 for the 14- and 22-feature datasets, respectively (see Table 2). After hyperparameter optimization with random search, the score for ROC_AUC was 0.721 for the 14-feature dataset, while the score for the 22-feature dataset was 0.718, as reported in Table 3. Support vector machines trained with 14 and 22 features had baseline scores of 0.712 and 0.718, respectively, as reported in Table 2. After hyperparameter tuning, the scores were 0.711 for the 14-feature dataset and 0.719 for the 22-feature dataset, as shown in Table 3.

We tuned and validated 11 voting classifiers assembled from all possible combinations of four tuned classifiers, namely decision tree, neural network, support vector machine, and random forest. All voting classifiers were set to a soft vote to enable a probability prediction, which is necessary for obtaining a ROC_AUC score. A hard vote approach forces the classifier to make a prediction based on a majority vote. In contrast, a soft vote approach has the voting classifier make a prediction based on an arg max of the predicted probabilities from each classifier. For a voting classifier, the two most crucial parameters to configure are the estimators or classifiers and the weights assigned to each estimator. To build the voting classifiers, we use the first best hyperparameter configurations for each of the base four classifiers (i.e., decision tree, neural network, support vector machine, and random forest) to create all possible combinations of two, three and four estimators. These combinations were passed as an array of estimators to the ensemble. The details of the search space are shown in Table 3; however, each estimator takes a weight of 1, 2, 3 or 4 in all voting classifiers. We adopted a grid search approach to optimize the hyperparameters with both versions of the datasets (i.e., 14 and 22 features) on each one of the 15 voting classifiers. Once we had optimized the ensembles, we again conducted 10-fold cross-validation against the 14- and 22-feature datasets; the results of the validation are summarized in Table 3. The voting classifier built with the tuned decision tree and random forest classifiers reported the highest ROC_AUC score: 0.73 against the 22-feature dataset, as reported in Table 5. The hyperparameter configuration for the best classifier can be found in the supplementary code (Supplementary Material).

## 4. Discussion

General practitioners in the field are required to decide whether a patient has a flu-like disease or a severe acute respiratory infection. This prediction is based exclusively on symptoms recorded in the raw dataset described at the beginning of Section 3. Plotting the receiving operating characteristic curve or calculating the area under the curve is not feasible for predictions made by general practitioners. However, we can calculate from the original dataset the sensitivity, specificity and precision, which are around 0.3686, 0.6675, and 0.3747, respectively.

By observing the sensitivity values from Table 3, we can see that the support vector machine on the 22 features has the highest score (0.788), but at the same time, 43.1% of negative patients are predicted to be infected with SARS-CoV-2. The false positive rate creates a false alarm for a significant number of non-infected patients. We are equally as concerned with causing the lowest number of false alarms possible as we with detecting the highest possible number of infected people. Somehow, we need to measure the trade-off between these two metrics. The first approach is to use the ROC_AUC value. After that, we wished to decide which classifier (in case of a tie with ROC_AUC) is the best. Our approach was to use the F1 metric. Table 5 summarizes the scores for the model that presented the highest ROC_AUC and F1 values, which was the ensemble of DT and RF with 22 features. The mean ROC_AUC score was 0.728, with a standard deviation of 0.042, with a mean sensitivity of 0.752 and a mean specificity of 0.609.

It is paramount for us to make clear that under no circumstances are we aiming to replace actual diagnostic tests like the RT-PCR test with a classifier, nor do we claim to outperform the assessment of primary care practitioners. Our main objectives are:

- To shed light on the problem of detecting COVID-19 with machine learning tools that help make sense of all the data being produced during this emergency.
- As health services everywhere are facing an overwhelming increase in demand from patients infected with COVID-19 in addition to other patients, some governments and health authorities are exploring non-conventional approaches to reduce the number of infections. Some of these require the population to take matters into their own hands and do their part to protect themselves in order to avoid the necessity of resorting to health services in the first place. This study aims to serve as one of these non-conventional approaches, as the approach can be made available for individuals as a machine learning tool capable of performing an almost immediate assessment of the probability of being infected with SARS-CoV-2.
- A machine learning model trained with the correct dataset could become a powerful aid for primary care practitioners, as it has applications as a tool for assisted decision-making.

It is also necessary to state that although this particular study may be related to an extent to other works in CADx, we do not present a CADx system. The classifiers presented in this study aim to automate the initial assessment of potential COVID-19 patients with predictions only on the basis of self-reported symptoms; the results are also intended to establish a baseline for future research on our dataset for the topic of symptoms-based COVID-19 detection. We cannot emphasize enough that our results show that the classifiers presented in this study are not a substitute for a RT-PCR test; instead, we argue that an ML-powered automated assessment may help optimize the use of the limited testing capacity of Mexican public health authorities. The practical applications of this work will be the subject of further research, as it could be used as a way to track and study the spread of the pandemic in large populations, and not only from the perspective of individuals.

## 5. Conclusions

Experienced primary care practitioners (i.e., physicians) often reach accurate diagnoses based on signs, symptoms, and past experiences. The best classifier reported in this document outperforms anamnesis from health professionals, as recorded in the dataset. We have to clarify that while our best model presents a higher sensitivity and precision, it also underperforms (by less than 10%) with respect to specificity. However, as per the results reported in this study, we believe that a symptoms-based CADx tool is still not viable with the classifiers reported in this study, and could not replace actual RT-PCR tests. One limitation of this study is that, ironically, the pandemic has made it too difficult to conduct a proper evaluation with patients measuring the efficacy and usefulness of this approach. Another aspect of this tool is that it relies heavily on patients’ ability to identify and report symptoms. Further evaluation is necessary to better understand patients’ ability to identify and report symptoms, as well as the characteristics of an assessment tool (i.e., mobile app or web app) for mitigating any possible problems that may arise in a self-reporting scenario. While we had enough instances in our dataset to use conventional ML algorithms (as opposed to Deep Learning) to train classifiers, we believe that a larger dataset would enable us to try Deep Learning and possibly improve the results. With supply chains strained by the high material demands for making more tests, and an increasing number of suspected COVID-19-positive subjects, an effective prioritization of who has access to tests for detection of COVID-19 is necessary. This paper contributes a machine learning model that detects infected COVID-19 patients with an average sensitivity of 0.752, which could assist in this task. Furthermore, it establishes a baseline for future research in this topic (i.e., symptoms-based, ML-powered detection of COVID-19) on the contributed dataset or a similar dataset. Another contribution of this study is the revision and aggregation of two COVID-19 datasets into one, which is available for other data scientists to explore and contribute to this problem.

## Figures and Tables

**Table 1:** Feature importance from the 22 independent variables in the dataset and ROC_AUC scores from fitting the dataset to a baseline random forest classifier by progressively including features according to ranking.

Ranking	Gini-Importance	ROC_AUC	Feature Name	Behavior (ROC_AUC)
1	0.0829	0.6328	FEVER	baseline
2	0.0616	0.6510	COUGH	+
3	0.0598	0.6396	SUDDEN_ONSET_OF_SYMPTOMS	-
4	0.0579	0.6841	KNOWN_COVID_CONTACT	+
5	0.0573	0.6947	ODYNOPHAGIA	+
6	0.0558	0.6976	RHINORRHEA	+
7	0.0535	0.6713	IRRITABILITY	-
8	0.0519	0.6826	CHEST_PAIN	+
9	0.0517	0.6664	CEPHALEA	-
10	0.0516	0.6552	CHILLS	-
11	0.0501	0.6687	DIARRHEA	+
12	0.0478	0.6764	FATIGUE_LOSS_APPETITE	+
13	0.0443	0.6905	DYSPNEA	+
14	0.0435	0.7005	MYALGIA	+
15	0.0425	0.6966	ARTHRALGIA	-
16	0.0403	0.6945	CONJUNCTIVITIS	-
17	0.0370	0.7010	ABDOMINAL_PAIN	+
18	0.0293	0.7122	POLYPNEA	+
19	0.0275	0.7064	VOMIT	-
20	0.0235	0.7098	ANOSMIA	+
21	0.0185	0.6864	DYSGEUSIA	-
22	0.0115	0.6985	CYANOSIS	+

**Table 2:** Baseline scores for all classifiers with 14 and 22 features.

CLASSIFIER NAME	FEATURES	SESITIVITY	SPECIFICITY	PRECISION	F1	ACCURACY	MEAN_AUC	STD_AUC
DECISION TREE	14	0.536	0.699	0.642	0.605	0.617	0.622	0.064
NEURAL NETWORK	14	0.699	0.631	0.655	0.661	0.665	0.712	0.040
RANDOM FOREST	14	0.636	0.674	0.661	0.653	0.655	0.680	0.054
SUPPORT VECTOR MACHINE	14	0.734	0.618	0.659	0.670	0.676	0.712	0.050
VOTING DT & NN	14	0.611	0.661	0.644	0.632	0.636	0.672	0.052
VOTING DT & RF	14	0.590	0.669	0.641	0.624	0.630	0.655	0.058
VOTING DT & SVM	14	0.620	0.649	0.639	0.632	0.634	0.670	0.052
VOTING DT, NN & RF	14	0.607	0.670	0.648	0.635	0.639	0.678	0.055
VOTING DT, NN & SVM	14	0.631	0.649	0.643	0.637	0.640	0.692	0.053
VOTING DT, NN, SVM & RF	14	0.636	0.656	0.647	0.644	0.646	0.691	0.052
VOTING DT, SVM & RF	14	0.612	0.662	0.644	0.633	0.637	0.680	0.054
VOTING NN & RF	14	0.673	0.668	0.670	0.669	0.670	0.702	0.048
VOTING NN & SVM	14	0.703	0.629	0.656	0.662	0.666	0.716	0.040
VOTING NN, SVM & RF	14	0.684	0.641	0.656	0.660	0.662	0.707	0.050
VOTING SVM & RF	14	0.672	0.652	0.660	0.661	0.662	0.698	0.050
DECISION TREE	22	0.556	0.668	0.629	0.605	0.612	0.606	0.039
NEURAL NETWORK	22	0.649	0.643	0.647	0.643	0.646	0.690	0.046
RANDOM FOREST	22	0.666	0.631	0.645	0.645	0.648	0.703	0.043
SUPPORT VECTOR MACHINE	22	0.743	0.609	0.656	0.668	0.676	0.718	0.048
VOTING DT & NN	22	0.585	0.642	0.622	0.610	0.613	0.679	0.050
VOTING DT & RF	22	0.585	0.638	0.620	0.607	0.611	0.681	0.045
VOTING DT & SVM	22	0.595	0.635	0.622	0.612	0.615	0.688	0.049
VOTING DT, NN & RF	22	0.619	0.661	0.647	0.638	0.640	0.696	0.048
VOTING DT, NN & SVM	22	0.625	0.644	0.639	0.633	0.635	0.700	0.046
VOTING DT, NN, SVM & RF	22	0.658	0.649	0.653	0.651	0.654	0.708	0.046
VOTING DT, SVM & RF	22	0.601	0.643	0.630	0.618	0.622	0.696	0.043
VOTING NN & RF	22	0.666	0.616	0.635	0.637	0.641	0.708	0.050
VOTING NN & SVM	22	0.692	0.616	0.644	0.649	0.654	0.713	0.049
VOTING NN, SVM & RF	22	0.699	0.616	0.647	0.652	0.657	0.720	0.046
VOTING SVM & RF	22	0.702	0.623	0.652	0.657	0.662	0.725	0.043

**Table 3:** Results after hyperparameter optimization for all classifiers with 14 and 22 features.

CLASSIFIER NAME	NUMBER OF FEATURES	SESITIVITY	SPECIFICITY	PRECISION	F1	ACCURACY	MEAN_AUC	STD_AUC
DECISION TREE	14	0.620	0.667	0.651	0.639	0.643	0.686	0.038
NEURAL NETWORK	14	0.726	0.619	0.658	0.665	0.673	0.721	0.044
RANDOM FOREST	14	0.738	0.630	0.667	0.678	0.684	0.723	0.046
SUPPORT VECTOR MACHINE	14	0.710	0.639	0.664	0.670	0.674	0.711	0.047
VOTING DT & NN	14	0.712	0.630	0.659	0.667	0.671	0.710	0.037
VOTING DT & RF	14	0.716	0.642	0.669	0.675	0.679	0.719	0.046
VOTING DT & SVM	14	0.707	0.633	0.660	0.665	0.670	0.708	0.047
VOTING DT, NN & RF	14	0.715	0.628	0.661	0.666	0.672	0.721	0.046
VOTING DT, NN & SVM	14	0.718	0.631	0.661	0.669	0.674	0.722	0.045
VOTING DT, NN, SVM & RF	14	0.717	0.634	0.664	0.671	0.675	0.722	0.041
VOTING DT, SVM & RF	14	0.726	0.631	0.664	0.673	0.678	0.719	0.047
VOTING NN & RF	14	0.733	0.625	0.662	0.674	0.679	0.724	0.045
VOTING NN & SVM	14	0.716	0.634	0.663	0.670	0.675	0.718	0.038
VOTING NN, SVM & RF	14	0.722	0.631	0.663	0.671	0.677	0.721	0.047
VOTING SVM & RF	14	0.733	0.633	0.668	0.677	0.683	0.721	0.048
DECISION TREE	22	0.747	0.552	0.631	0.622	0.650	0.693	0.040
NEURAL NETWORK	22	0.682	0.638	0.654	0.655	0.660	0.718	0.051
RANDOM FOREST	22	0.729	0.607	0.652	0.660	0.668	0.730	0.047
SUPPORT VECTOR MACHINE	22	0.788	0.569	0.647	0.659	0.678	0.719	0.048
VOTING DT & NN	22	0.708	0.626	0.656	0.662	0.667	0.726	0.042
VOTING DT & RF	22	0.742	0.601	0.652	0.661	0.672	0.730	0.044
VOTING DT & SVM	22	0.763	0.591	0.652	0.665	0.677	0.726	0.040
VOTING DT, NN & RF	22	0.714	0.623	0.657	0.663	0.669	0.722	0.050
VOTING DT, NN & SVM	22	0.712	0.618	0.653	0.658	0.665	0.721	0.041
VOTING DT, NN, SVM & RF	22	0.727	0.619	0.658	0.665	0.673	0.729	0.045
VOTING DT, SVM & RF	22	0.739	0.608	0.655	0.665	0.674	0.728	0.046
VOTING NN & RF	22	0.713	0.618	0.653	0.660	0.666	0.728	0.049
VOTING NN & SVM	22	0.721	0.618	0.656	0.664	0.670	0.726	0.050
VOTING NN, SVM & RF	22	0.726	0.622	0.660	0.667	0.674	0.730	0.048
VOTING SVM & RF	22	0.742	0.608	0.656	0.665	0.675	0.729	0.048

**Table 4:** Search space as defined for each classifier.

Decision Tree	
**Hyperparameters**	Range of values
**criterion**	[‘Gini’, ‘entropy’]
**splitter**	[‘best’, ‘random’]
**max_depth**	[50 values equally distributed between 2 and 103 (inclusive)]
**min_samples_split**	[2, 3, 4, 5, 6, 7, 8, 9, 10]
**min_samples_leaf**	[2, 3, 4, 5, 6, 7, 8, 9, 10]
**max_features**	[0.1, 0.2, 0.3, 0.4, 0.5, 0.6, 0.7, 0.8, 0.9, 1.0]
**ccp_alpha**	[0.0, 0.1, 0.2, 0.3, 0.4, 0.5, 0.6, 0.7, 0.8, 0.9, 1.0]
**Search space:**	1782000
**Random Forest**	
**n_estimators**	[500 equally distributed values between 2 and 2003 (inclusive)]
**criterion**	[‘Gini’, ‘entropy’]
**max_depth**	[50 equally distributed values between 2 and 103 (inclusive)]
**min_samples_split**	[2, 3, 4, 5, 6, 7, 8, 9, 10]
**min_samples_leaf**	[2, 3, 4, 5, 6, 7, 8, 9, 10]
**# max_features**	[‘auto’, ‘sqrt’, ‘log2’, None]
**max_features**	[0.1, 0.2, 0.3, 0.4, 0.5, 0.6, 0.7, 0.8, 0.9, 1.0]
**bootstrap**	[True, False]
**ccp_alpha**	[0.0, 0.1, 0.2, 0.3, 0.4, 0.5, 0.6, 0.7, 0.8, 0.9, 1.0]
**Search space:**	3564000000
**Support Vector Machine**	
**C**	[40 equally distributed values between 0 and 2 (inclusive)]
**kernel**	[‘linear’, ‘poly’, ‘rbf’, ‘sigmoid’]
**degree**	[1, 2, 3, 4, 5, 6]
**gamma**	[100 equally distributed values between 0.000000001 and 0.999 (inclusive)]
**coef0**	[200 equally distributed values between 0 and 1 (inclusive)]
**shrinking**	[True, False]
**Search space:**	38400000
**Neural Network**	
**hidden_layer_sizes**	[tuples of 1, 2 and 3 layers each with 15, 50, 80, 100 and 200 neurons]
**activation**	[‘identity’, ‘logistic’, ‘tanh’, ‘relu’]
**solver**	[‘lbfgs’, ‘sgd’, ‘adam’]
**alpha**	[500 equally distributed values between 0.00001 and 0.001 (inclusive)]
**learning_rate**	[‘constant’, ‘invscaling’, ‘adaptive’]
**learning_rate_init**	[20 equally distributed values between 0.00001 and 0.01 (inclusive)]
**power_t**	[10 equally distributed values between 0.1 and 1.0 (inclusive)]
**shuffle**	[True, False]
**Search space:**	1116000000
**Voting Classifier (Two Votes)**	
**estimators**	[6 different combinations of two estimators using the best DT, NN, SVM and RF]
**weights**	[16 different tuples of two integer values between 1 and 4 (inclusive)]
**Search space:**	96
**Voting Classifier (Three Votes)**	
**estimators**	[4 different combinations of two estimators using the best DT, NN, SVM and RF]
**weights**	[64 different tuples of three integer values between 1 and 4 (inclusive)]
**Search space:**	256
**Voting Classifier (Four Votes)**	
**estimators**	[1 tuple mad of all four estimators: DT, NN, SVM and RF]
**weights**	[256 different tuples of four integer values between 1 and 4 (inclusive)]
**Search space:**	256

**Table 5:** Results from the best classifier.

Metric	22 Features
accuracy	0.681
classifier	voting DT & RF
F1	0.670
mean_auc	0.728
precision	0.660
sensitivity	0.752
specificity	0.609
std_auc	0.042

## Data Availability

The dataset was made available online at https://github.com/rMartine/covid19 (accessed on 1 February 2021).

## Supplementary Materials

Python code is available online at https://github.com/rMartine/covid19; last accessed on 5 February 2021.
